# Optimisation of Design and Manufacturing Parameters of 3D Printed Solid Microneedles for Improved Strength, Sharpness, and Drug Delivery

**DOI:** 10.3390/mi12020117

**Published:** 2021-01-22

**Authors:** Sophia N. Economidou, Cristiane P. Pissinato Pere, Michael Okereke, Dennis Douroumis

**Affiliations:** 1 Medway School of Pharmacy, University of Kent, Medway Campus, Central Avenue, Chatham Maritime, Chatham, Kent ME4 4TB, UK; cristianepissinato@gmail.com; 2 Department of Engineering Science, University of Greenwich, Kent ME4 4TB, UK; M.I.Okereke@greenwich.ac.uk; 3 CIPER Centre for Innovation and Process Engineering Research, Kent ME4 4TB, UK

**Keywords:** microneedles, 3D printing, stereolithography, optimisation, inkjet coating

## Abstract

3D printing has emerged as a powerful manufacturing technology and has attracted significant attention for the fabrication of microneedle (MN)-mediated transdermal systems. In this work, we describe an optimisation strategy for 3D-printed MNs, ranging from the design to the drug delivery stage. The key relationships between design and manufacturing parameters and quality and performance are systematically explored. The printing and post-printing set parameters were found to influence quality and material mechanical properties, respectively. It was demonstrated that the MN geometry affected piercing behaviour, fracture, and coating morphology. The delivery of insulin in porcine skin by inkjet-coated MNs was shown to be influenced by MN design.

## 1. Introduction

The transdermal route offers significant benefits over oral and injection-based drug delivery strategies as it yields higher bioavailability [1], surpasses metabolic systems, is minimally invasive, and can be self-administered. Transdermal drug delivery systems have gained increased attention since the introduction of microneedles (MNs), which are arrays of microsized needles that painlessly disrupt the skin barrier to transport molecules directly into the dermis. MNs can enable drug delivery through multiple approaches mediated by solid (‘poke and patch’ method), solid coated, hollow, and dissolvable MNs [2]. 

Coated MNs are an appealing approach, since they have enabled the delivery of macromolecules, proteins, nanomedicines, DNA, and vaccines with fast drug release rates owed to the fast dissolution of the thin film coatings in the skin [3,4,5,6]. Moreover, unlike dissolvable MNs, the drug loading process is independent from the MN manufacturing process. This translates to a significant advantage in regard to mechanical performance, since the amount of drug loaded to the array does not affect the strength and piercing ability of the MNs [7]. This independence also endows the production stage with versatility as blank MN arrays from the same batch can be loaded with different therapeutic molecules based on product demand [8]. It has also been documented that preserving actives in a solid state may increase stability, prolonging their shelf life [9,10].

For the development of reliably coated MN systems, the adoption of advanced coating techniques is imperative. Amongst the key criteria that render a coating process effective are uniformity and reproducibility, accuracy so the drug is only deposited on the MNs, low process temperatures to avoid drug degradation, and a strong bonding of the coating film on the MN surface to reassure that drug will not remain on the skin surface upon piercing [11]. Numerous methods have been applied for this purpose, such as immersion of the whole patch [12], drop coating [13], and spray coating [14], which are prone to allow contamination of the MN substrate by the coating material. The prominent coating techniques that reassure localized coating formation exclusively on the MN bodies with no drug deposition on the substrate, are dip-coating and inkjet printing [7]. Dip-coating has had numerous applications on MN coating [15,16,17] because of its simplicity; however, issues regarding the accuracy of the coated drug amount have emerged. On the other hand, inkjet printing is a reliable, reproducible, and accurate coating method that enables spatially controlled film creation and has been successfully applied on MNs [18,19]. However, the effect of MN geometry on the morphology of the inkjet-coated films has not yet been fully understood.

MNs are typically manufactured via micromoulding techniques. However, recently the technology of 3D printing has revolutionised yet another scientific field, with research on the area of 3D printed MNs to have attracted extensive scientific and industrial attention [20]. Laser Stereolithography (SLA), a vat photopolymerization technology, has been proposed for the fast and cost-effective production of MNs that have been used for the delivery of insulin, anticancer drugs, and model dyes [21,22,23,24]. These studies demonstrate the suitability of 3D printed MNs for a range of applications, nonetheless a systematic study of the key challenges and critical development aspects for optimisation is lacking. 3D printing quality is notoriously sensitive to manufacturing parameters, and an application as demanding as the MNs requires thorough knowledge about the relationships between parameters and quality features.

In this work, we correlate design, printing, and post-printing parameters with MN quality and performance, in an attempt to build and deepen the knowledge on the impact of key design and manufacturing aspects on the MN function. The effect of printing angle and post-printing curing conditions on sharpness and material mechanical properties of SLA-printed MNs is examined. We hypothesize that the MN shape affects the coating morphology and hence its dissolution into the skin. Hence, we seek to identify the key geometrical parameters that affect the in vitro drug release profiles generated from 3D printed MNs, coated via a precise piezoelectric inkjet coating technique. The outcome of this study will provide insight to inform design practices, aiming at optimising 3D printed MNs for tuneable and customisable drug delivery. 

## 2. Materials and Methods

### 2.1. Materials

The MNs were fabricated using the biocompatible Class I acrylic resin, Dental SG (Formlabs, GoPrint3D, North Yorkshire, UK). Insulin derived from bovine pancreas (10 mg mL^−1^) was acquired from Sigma-Aldrich (Gillingham, UK). Polyvinylpyrrolidone (PVP, Kollidon 17PF) and Poloxamer 407 (Pluronic F-127) were purchased from BASF (London, UK). All solvents were of analytical grades.

### 2.2. Computed Aided Design (CAD)

Three MN geometries were designed using the PTC Creo Computer Aided Design (CAD) software (PTC, Boston, MA, USA). Based on shape, the geometries were named ‘cone’, ‘pyramid’, and ‘spear’. Their nominal dimensions are displayed in Table 1.

The MNs were designed to protrude from a square patch of 15 mm × 15 mm × 0.5 mm in a symmetrical configuration of 7 rows and 7 columns, with a 1.85 mm of interspacing for all designs. Moreover, solid cylinders were designed in accordance with the requirements of the ASTM D695-15 protocol. The cylinders of ø12.7 mm and length 25.4 mm were intended for mechanical testing to investigate the effect of curing conditions on compressive properties.

### 2.3. Additive Manufacturing 

A Form2 (Formlabs, GoPrint3D, Ripon, North Yorkshire, UK), a 3D printer based on the technology of laser SLA, was employed to manufacture MN patches. Prior to printing, the virtual models were imported in the printer software, PreForm (Formlabs, Somerville, MA, USA), in order for the final printing file to be generated. The layer thickness was set at 50 μm and structure supports were manually added with a touchpoint size of 0.4 mm. A base was used to connect the supports with the printing platform for each MN array. The patches were rotated at 0°, 90°, and 45° to the printing platform to study the effect of the printing orientation on MN quality and dimensional accuracy. The code generated by the printing software was imported in the 3D printer that was equipped with a vat of unpolymerized Dental SG resin. The printed structures were submerged in an isopropyl alcohol bath to rinse away any unpolymerized resin residues. The printed structures were then cured in a UV-A heated chamber (MeccatroniCore BB Cure Dental station, GoPrint3D, Ripon, North Yorkshire, UK) at a wavelength of 405 nm. The cylinders for mechanical testing were subjected to 9 different curing regimes, each combining a preset curing time (30 min, 45 min, and 60 min) and chamber temperature (40 °C, 50 °C, and 60 °C). The combination of curing parameters that was found to optimize material mechanical behaviour was applied for the curing of MN patches.

### 2.4. Dental SG Compression Testing 

The effect of different curing conditions on the mechanical properties of the printing material was explored. The printed resin cylindrical specimens were subjected to compression testing, complying with the ASTM D695 – Standard Test Method for Compressive Properties of Rigid Plastics, international testing protocol. A Tinius Olsen universal testing machine was employed, equipped with a 25kN load cell and two flat, cylindrical test fixtures. The specimen was positioned at the center of the fixed test fixture while the driven one was moving downwards along the specimen longitudinal axis at a constant speed of 1.5 mm/min. Continuous force and displacement data were recorded for every specimen. The generated data were processed according to the offset curve method. True stress and true strain data were calculated along with the compressive moduli and yield strengths for all curing regimes. A total of 5 specimens were tested for each set of curing conditions.

### 2.5. Scanning Electron Microscopy (SEM)

The MN arrays were mounted onto aluminium stubs using a double-sided carbon adhesive tape (Agar scientific, UK). Each MN array was examined by SEM (Hitachi SU 8030, Japan) using a low accelerating voltage (1.0 kV). A low accelerating voltage was used to avoid electrical charges on the MNs. The images of the MNs were captured digitally from a fixed working distance (11.6 mm) using different magnifications. Processing the images and measurements were conducted through the software ImageJ (Fiji package) (NIH image, Bethesda, MD, USA). 

### 2.6. Preparation of Porcine Skin Samples

Full-thickness abdominal porcine skin was collected from a local slaughterhouse (Forge Farm Ltd., Kent, UK) and was then shaved using a razor blade. The fatty tissue below the abdominal area was removed using a scalpel. Samples of full-thickness skin tissue were sliced and used for the piercing tests. The remaining skin was pinned onto a polystyrene block and wiped with ethanol (70%). Samples of 1.0 ± 0.1 mm thickness were extracted by applying a Dermatome (Padgett Dermatome, Integra LifeTMSciences Corporation, Princeton, NJ, USA) at an angle of ±45°. The thickness of the skin was measured using a caliper and 20 mm × 20 mm tissue samples were cut using a scalpel. The skin samples were placed onto filter paper soaked in a small amount of saline phosphate buffer (pH 7.4) for 2 h. The Dermatome skin samples were employed for the in vitro release studies.

### 2.7. Piercing Tests in Porcine Skin

The 3D printed cone, pyramid, and spear MNs were subjected to piercing tests in porcine skin. A TA.HD plus Texture Analyser (Stable Micro Systems, Surrey, UK) equipped with a 5 kg of load cell was employed. The MN arrays were fixed on the moving probe of the machine using double-sided adhesive tape. Samples of full-thickness abdominal skin were placed on waxed petri dishes and were secured on the bottom, fixed probe of the machine. Continuous force and displacement measurements were recorded to identify the point of needle insertion. The speed of the moving probe was 0.01 mm/s and all experiments were repeated 5 times.

### 2.8. Microneedles (MN) Axial Force Mechanical Testing 

To evaluate the fracture strength of the cone, pyramid, and spear MNs, fracture tests were conducted employing a Tinius Olsen H25KS mechanical testing machine (Tinius Olsen TMC, Horsham, PA, USA), equipped with a 25 kN load cell and two flat, cylindrical test plates. The MNs were mounted on the moving probe of the machine using double-sided tape and were compressed against the unmoving flat steel plate until fracture. Continuous force and displacement measurements were recorded. The speed of the moving probe was 1 mm/s and each experiment was repeated 5 times.

### 2.9. Circular Dicroism (CD) 

Pure insulin and insulin-polymer formulations were developed at a ratio 5:1. The formulations were diluted to 1.0 mg mL^−1^ in deionized water and the spectra were recorded at 20 °C between 190 and 260 nm by CD (Chirascan, Applied Photophysics, UK) using a 0.1 mm polarization certified quartz cell (Hellma, Müllheim, Germany). Spectra were recorded using a step size of 1 nm, a bandwidth of 1 nm, and an acquisition time of 1 s. Four scans were recorded for each sample, averaged, and a corresponding spectrum of water was subtracted from each spectrum. 

### 2.10. Inkjet Printing on 3D Printed MNs 

The insulin-polymer solutions were deposited on the cone, pyramid, and spear MN lateral surfaces in the form of fine droplets using an inkjet printer (NanoPlotter II, Gesim, Germany), equipped with a piezo-driven dispenser (PicPip 300). All MN arrays were fixed on a metal stub at 45° relative to the dispenser. For each coating cycle, 10 dots of 2 droplets were dispensed along the longitudinal axis of each MN. The process was repeated for 32 jetting cycles for each side of the array (two lateral MN surfaces were coated) resulting in 5 IU (175 μg) of insulin per array. The solvent (deionised water) was allowed to evaporate through incubation for 24 h at room temperature.

### 2.11. In Vitro Release of Insulin Coated MNs

The delivery of insulin loaded on the MNs was studied, using abdominal porcine skin. The in vitro testing was carried out in Franz diffusion cells (PermeGear, Inc., Hellertown, PA, USA). Dermatomed skin samples were placed in phosphate buffered saline (PBS; pH 7.4) for 1 hour prior to experimentation. The MN arrays were inserted into the skin samples using manual finger pressure. The skin and MN array was mounted onto the donor compartment of a Franz diffusion cell. The temperature of the Franz cells was maintained at 37 °C using an automated water bath (Thermo Fisher Scientific, Newington, USA). Sample fractions (6–6.5 mL/h) were collected using an auto-sampler (FC 204 fraction collector, Gilson, Middleton, WI, USA) attached to the Franz diffusion cells. Statistical analysis for the drug release was performed by using a Mann–Whitney nonparametric test (InStat, GraphPad Software Inc., San Diego, CA, USA), where samples are considered to be statistically significant at *p* < 0.05.

### 2.12. High-Performance Liquid Chromatography (HPLC)

The amount of insulin collected from the receptor fluid was determined by High-Performance Liquid Chromatography (HPLC; Agilent Technologies, 1200 series, Cheshire, UK) equipped with a Phenomenex Jupiter 5u c18 300 Å, LC Column (250 mm × 4.60 mm, particle size 5 μm, Macclesfield, UK). The mobile phase consisted of water with 0.1% trifluoroacetic acid (TFA) and acetonitrile with 0.1% TFA (66:34 *v*/*v*), with a 1 mL min^−1^ flow rate. The column was equilibrated at 35 °C, the injection volume was 20 μL, and the eluent was analysed with a UV detector at 214 nm. The results were integrated using Chemstation^®^ software and the samples were analysed in triplicates.

## 3. Results

### 3.1. Additive Manufacturing

The three MN geometries, cone, pyramid, and spear, and the respective 7 × 7 symmetric patches were developed using an engineering design software. The virtual models were then ‘sliced’ and imported in the printer software where the printing parameters were selected. The layer thickness was set at 50 μm, which was the highest resolution obtainable for the specific material. Resolution is adversely proportional to the printing time for this technology, however the importance of high end-product definition was considered a priority over production time. The touchpoint size of 0.4 mm was found to allow subsequent removal of the supports without damaging the delicate MN structures. 

The 3D printer followed the standard building process of typical laser SLA. A building platform was submerged in a vat of unpolymerized photocurable resin and through an optical window, the laser ‘wrote’ on the resin, selectively polymerizing fine, coalescing roads and forming consecutive layers. After each layer, the printing platform moved upwards, detaching the printed structure from the optical window and allowing for a mechanical sweeper blade to recoat the surface with fresh unpolymerized resin. After completion, the MN patches were rinsed in isopropyl alcohol to remove unpolymerized resin residues. 

For the purpose of this study, we employed a commercial Class I biocompatible resin as the MN manufacturing material. The printed material, having been subjected to the post-printing washing and curing protocol described here, has been found to comply with ISO biocompatibility standards and thereby rendered biocompatible. The aforementioned testing, encompassing systemic injection (acute systemic toxicity), intracutaneous and implantation tests, concluded classification of the material as USP IV according to the United States Pharmacopeia and National Formulary (USP-NF) [25]. These findings coincide with other reports found in the literature [26,27]. The verified biocompatibility of the polymer is a prerequisite for the potential clinical applicability of the proposed MN patch. 

### 3.2. Optimisation of Post-Printing Curing Conditions for Enhanced Mechanical Properties

**Figure 1:**
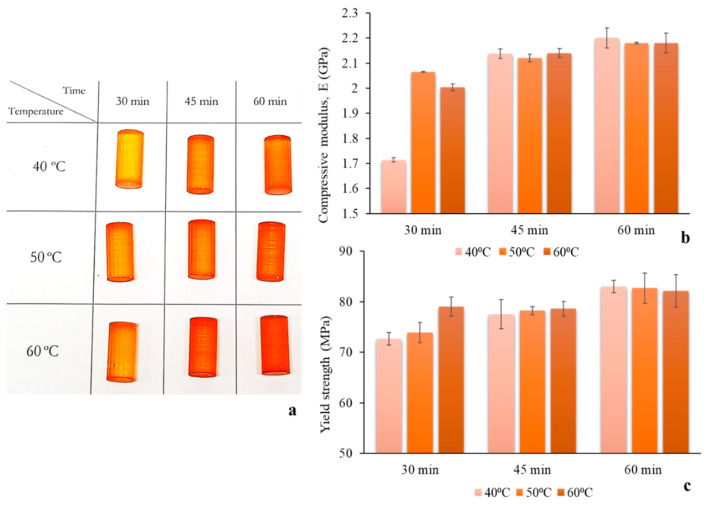
Optimisation of post-printing curing regime in respect to compressive mechanical properties: (**a**) Digital image of compression specimens for different curing settings, (**b**) compressive moduli, and (**c**) yield strengths obtained by specimens subjected to different curing regimes.

Prior to post-printing curing of MNs, the effect of curing conditions on the mechanical properties of the material was evaluated. It was hypothesised that the post-printing curing regime influences the cross-linking density, which has been documented to be closely associated with Young’s modulus and ultimate strength [28,29]. The aim of this study was to identify the curing settings that yield optimal material mechanical properties, thereby improving the mechanical behaviour of the MNs. As MNs are mainly subjected to compressive stresses along their longitudinal axis during application, the focus of this study was put on improving the compressive properties of the material. Hence, standard cylindrical specimens for compression testing [30] were designed and printed using the same material and settings as the MNs. Different curing regimes were imposed featuring varying durations of UV-A exposure and chamber temperature, in the respective ranges permitted by the manufacturer (30 to 60 min and 40 to 60 °C). Visual observation of the specimens revealed a gradual deepening of the colour with the increase of both exposure time and temperature (Figure 1a), which indicates an increasing degree of cross-linking [31]. 

During mechanical testing, it was found that the 25 kN load cell of the testing machine was not sufficient for the determination of ultimate strength. Instead, the yield strength was calculated. The exposure time was found to have a more pronounced and sustained effect on both properties than chamber temperature, with longer exposure times to lead to increased mechanical properties (Figure 1b,c). Indeed, the influence of chamber temperature was found to weaken with increasing exposure time and practically vanished for specimens exposed for 60 min. Both compressive modulus and yield strength maximum values, 2.2 GPa and 83 MPa respectively, were reached for curing at 40 °C and 60 min. The latter were employed as the standard post-printing curing regime for the duration of the present study. These findings suggest that optimising the post-printing curing regime is a crucial step for photopolymerization printed MNs, in the sense that a mechanically strong material reduces the chances of MN failure during application and increases confidence in the system.

### 3.3. Optimisation of Printing Angle 

After optimisation of the post-printing curing conditions, the effect of the printing angle on MN quality and dimensional accuracy was studied. The starting hypothesis of this study was based on two arguments: (1) The printing angle has an effect on the shape of the laser focal point as it focuses on the resin, thereby changing the sharpness of detail definition and (2) depending on the printing angle, the surface area in contact with the optical window changes. The latter influences the magnitude of the mechanical stress induced by the printing process, particularly when upon completion of each layer the structure is detached from the optical window by being drawn upwards. Therefore, reducing the cross-sectional surface in the *z* axis is anticipated to reduce the in-process mechanical stress which, if extended, can introduce severe defects and printing failures.

**Figure 2:**
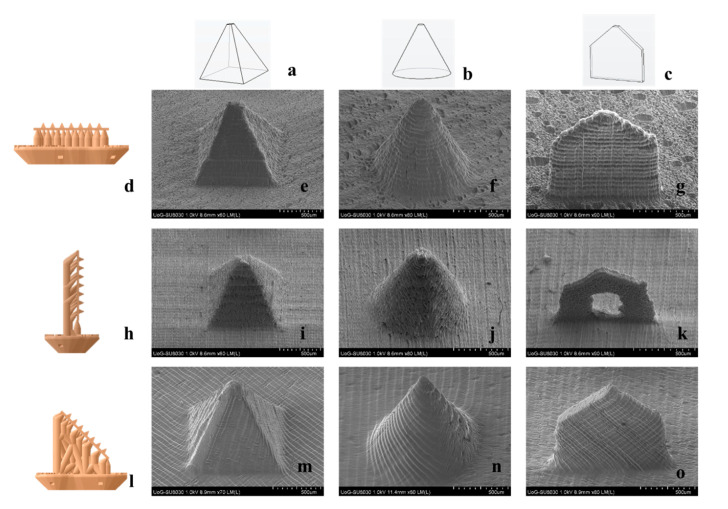
Optimisation of the printing angle; (**a**,**b**,**c**) CAD captions; (**d**,**h**,**l**) virtual models of supported MN arrays for 0°, 90° and 45°, where the bottom of the base is adhered to the printing platform; (**e**,**f**,**g**) SEM captions of MNs built at 0°; (**i**,**j**,**k**) SEM captions of MNs built at 90°; and (**m,n,o**) SEM captions of MNs built at 45°.

Three printing angles were applied, by rotating the MN arrays at 0°, 90°, and 45° in respect to the printing platform. Figure 2 features SEM images of MNs printed at different angles. The effect of the angle on MN quality is visually obvious, with MNs built at 0° (Figure 2e–g) and 90° (Figure 2i–k) to appear blunter. Noticeably, the spear built at 90° (Figure 2k) showed significant structural defects. This is a typical example of in-process developed stresses that cause detrimental flaws to the point of printing failure. When built at 90°, the surfaces in contact with the optical window are the consecutive cross-sectional areas along the fine thickness of the spear, which are not able to withstand the mechanical stress during detachment. Nonetheless, at 45° the quality seems to improve significantly and the MNs appear sharper with no structural manufacturing failures detected. 

**Figure 3:**
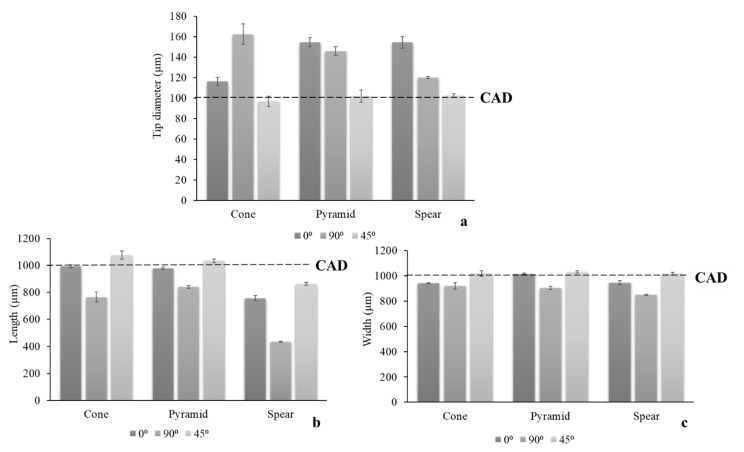
Dimensional discrepancies of printed MNs in respect to printing angle; (**a**) tip diameter; (**b**) length at the longitudinal axis; and (**c**) width at the base.

To further elucidate the visually distinguishable impact of a printing angle on MN quality, the dimensional discrepancies of the printed structures in comparison to the original CAD dimensions (Table 1) were quantified. Three critical parameters, tip diameter, length at the longitudinal axis, and width at the base, were measured for all angles (Figure 3). 

In accordance with visual evidence, printing at 45° yielded the optimal dimensional fidelity to the nominal CAD dimensions. The parameter that was found to be more sensitive to the printing angle was the MN tip diameter, wherein the larger dimensional discrepancies were detected. At the same time, it is considered the most significant parameter, as increased MN sharpness is crucial for clinical application and is associated with the minimisation of pain [32]. MN designs were not equally prone to structural defects, with spear being more susceptible due to its fine thickness. 

The results suggest that the contact area with the optical window plays a prominent role, however it should not be considered solely. Τhis can be observed in the case of 45° and 90°, where the area in contact with the optical window during printing is similar. Nonetheless, the dimensional discrepancies between both designs are large, leading to the conclusion that the amount of support that has been printed for each new printing cycle is critical. At 45° a sufficient amount of support structure has already been printed prior to initiating the fabrication of the MN patch. This provides a wider adhesion area to the building platform and hence a larger area for stress distribution. On the other hand, at 90°, the supports are printed simultaneously with the MN. The smaller support area for each new layer leads to increased mechanical stresses imposed on the MN during printing, and thus defective prints.

The findings of this study suggest that the effect of printing orientation for 3D printing technologies that involve a recurring mechanical detachment of the structure from a surface intermediately between layers, is generally underplayed. The hypothesis that the build-up of mechanical stresses causes geometrical discrepancies and often detrimental structural flaws, was validated. It was also demonstrated that minimising the *z* axis cross sectional area should also be combined with careful thought of support placement, so that the MN is adequately supported during manufacturing. The optimized printing angle of 45°, which was used for the remaining of the present work, is in agreement with a respective study on hollow MNs [33].

### 3.4. Piercing Tests

The 3D printed cone, pyramid, and spear MNs were subjected to piercing testing using porcine skin to elucidate the effect of design on the maximum force required for successful insertion. The symmetrical 7 × 7 configuration of the arrays is a prerequisite for a uniform distribution of the externally applied force, permitting the calculation of the piercing force required for individual needles, so that the results are readily transferable to larger arrays. 

**Figure 4:**
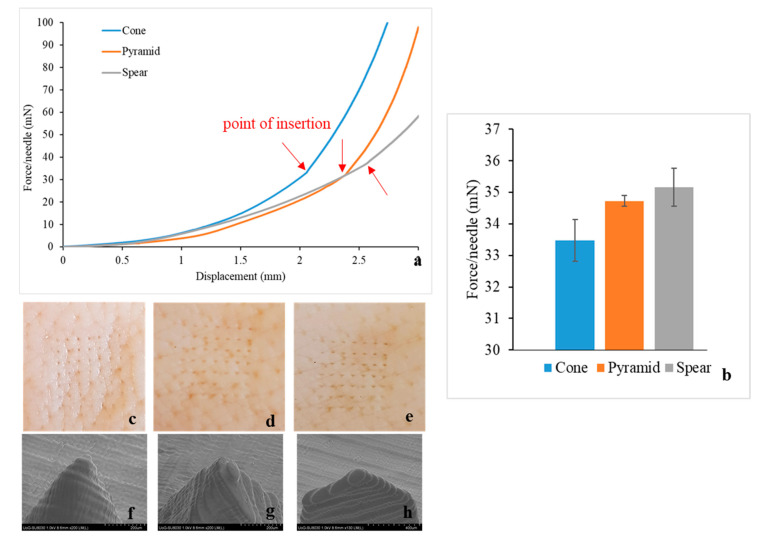
Piercing tests in porcine skin; (**a**) force/needle vs displacement data for all designs; (**b**) insertion force/needle; digital images of the porcine skin samples after piercing with (**c**) cone, (**d**) pyramid, and (**e**) spear MNs; and (**f**,**g**,**h**) post-testing SEM images of the MN tips.

All MN designs demonstrated similar piercing behaviour (Figure 4a), leaving visually observable pores on the skin after testing (Figure 4c–e). No MN failure was reported during experimentation. Thereafter, the MNs were separated from the skin tissue and examined via SEM, to investigate for geometrical alterations or damage around the tip area caused by the insertion (Figure 4f–h). No MN blunting, flattening, abrasion, or bending of the tip was observed and the tip diameter remained intact.

In accordance to findings described in the literature [21,22,34,35], piercing is not identified as a single event, but rather as a series of gradual tears of the skin as the MN proceeds further through the tissue. This explains the constant change of slope prior to the insertion point. Skin is considered to behave as an elastic material in the load range of 0–20 N [36], hence curve linearity (constant slope) would be anticipated if this was simple skin deflection. On the contrary, the slope changed constantly, indicating progressive tearing of the skin, until a sharp drop of load or an abrupt change in slope indicated the point of insertion. Then, the MNs remained inserted in the tissue and further increase of force was attributed to the compression of the skin-MN system.

All MN designs required minimal insertion forces. The insertion force was found to be affected by the MN design, with the cone requiring the lowest and spear the highest externally applied load (Figure 4b). Aoyagi at al. demonstrated that for single needles, the tip angle is proportional to puncture force [37]. The spear geometry was designed with a tip angle of 97°, as opposed to cone and pyramid where the tip angle was 48.5° (Table 1), which explains that spear MNs required the highest force for piercing. Moreover, regarding the cone and pyramid that feature identical tip angles, the magnitude of the MN surface area is anticipated to affect MN insertion, in the sense that friction forces develop during gradual penetration. The lateral pyramid surface is larger by 21% compared to the respective cone surface [21], which justifies that the pyramid MNs require higher forces for skin insertion.

### 3.5. MN Axial Force Mechanical Testing 

**Figure 5:**
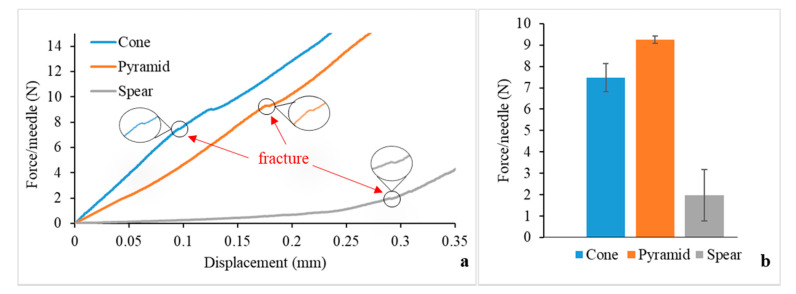
MN fracture testing: (**a**) Force vs. displacement curve, featuring magnified captions at the fracture points and (**b**) fracture forces.

The fracture strength of the MNs was examined, in order to assess the possibility of a fracture during clinical application and to compare the mechanical behaviour of the designs. The force-displacement data obtained from the axial force mechanical testing are displayed in Figure 5a. The point of fracture is identified by an apparent discontinuity of the curve. For the cone and pyramid designs, the slope is mainly constant until the fracture force is reached, meaning that in this load range the MNs can withstand the forces preserving their structural integrity without significant plastic (permanent) deformations. On the other hand, the spear design generated a curve with a changing slope before fracture, which can be attributed to bending. Indeed, these findings are in agreement with visual observations during testing; while the cone and pyramid designs failed at the direction parallel to the loading axis with an initial failure of the tip, the spear design failed at the base due to bending. Hence, the fracture force for the spear MNs was found to be lower than the pyramid and cone ones (Figure 5b). Nevertheless, the fracture forces for all designs (reported in N) were significantly higher than the respective forces required for piercing (reported in mN).

### 3.6. Safety Index

The results derived from the piercing and fracture testing can provide valuable insight regarding the safety of the MNs upon application. The margin of safety has been proposed by Prausnitz [38] and is calculated by the following equation:

(1) $$margin\;of\;safety=\frac{F_{fracture}}{F_{insertion}}$$

where *F_fracture_* and *F_insertion_* are the fracture and insertion forces, respectively.

Typically, the value of the margin needs to be higher than one, however the reliability of the MN system increases with higher values. The margins of safety calculated by Equation (1) for the cone, pyramid and spear designs are 223, 266, and 55, respectively. The spear design exhibits the lowest value as anticipated, however all margins of safety are well above 1, which is promising evidence that the MNs will be safe for clinical application.

### 3.7. Circular Dichroism

For the purpose of this work, two polymers were chosen as insulin carriers to maintain insulin stability in the solid state. Insulin-polymer formulations were developed, consisting of insulin and polymer excipients at a ratio of 5:1 (wt/wt). The polymers chosen were poloxamer and PVP, which have been both approved for parenteral delivery by the U.S. Food and Drug Administration (FDA) [39,40]. 

**Figure 6:**
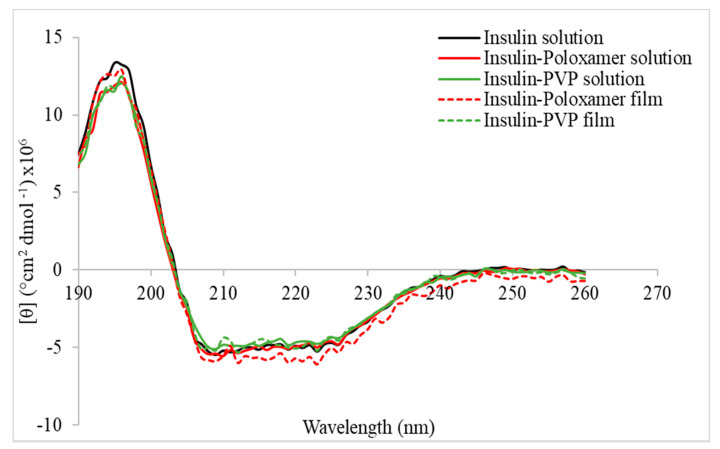
CD spectra for insulin and insulin-polymer carriers.

Prior to coating the MNs, the effect of the polymer carriers on the insulin molecule was assessed by means of CD. The study was conducted on both solutions and films for both carriers. Figure 6 depicts the far UV CD spectra of insulin and insulin-polymer solutions and films. All spectra showed two double minima at 210 and 225 nm, which is characteristic for a-helix predominant proteins [41]. A slight decrease in Molar ellipticity is shown for the insulin-poloxamer solution and is further intensified for the film, indicating a lower a-helical content. However, the presence of PVP seems to induce an increase of Molar ellipticity for both solution and dried forms, compared to the poloxamer-insulin formulation. This is an indication of increased a-helix content, implying that the PVP has a protective action on the insulin molecule in a solution as well as in the solid state, compared to the poloxamer. Indeed, the Molar ellipticity spectrum of the pure insulin solution coincides with the ones recorded for the PVP formulation in both solution and dried forms.

### 3.8. Inkjet Coating 

**Figure 7:**
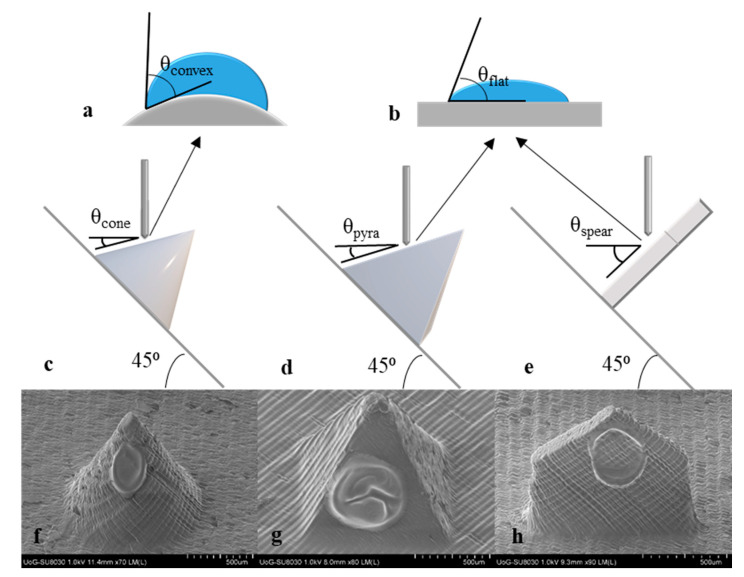
Inkjet coating; (**a**,**b**) static contact angle of inkjet droplet upon impingement on the MN surface for convex (cone) and flat (spear and pyramid) surfaces; (**c**,**d**,**e**) scheme of the coating configuration for cone, pyramid, and spear MNs; and (**f**,**g**,**h**) SEM images of the coated MNs.

Insulin-polymer coatings were deposited on the surfaces of the 3D printed MNs using an inkjet printing piezoelectric dispenser. The process parameters, namely the applied voltage and pulse duration were optimised previously [18], so that each droplet had a volume of 300 pL. The consecutive coating cycles and the coalesced droplets created uniform coating films on the MN surfaces, as illustrated in Figure 7f–h. The absence of satellite droplets on the MN substrate asserts the high accuracy of the system and is a good indication that no drug losses occured.

In the framework of this study, it was hypothesised that MN geometry influences coating morphology and thereby, dissolution. To get a better understanding of this hypothesis, the process of droplet impingement and spreading on the MN surface was examined. Upon impact on a surface, a droplet spreads radially and its spreading behaviour typically depends on impinging velocity, liquid properties, and impact surface [42]. The surface influences droplet shape and spreading not only in terms of material wettability (i.e., being hydrophilic or hydrophobic), but also through its geometry. Factors such as surface curvature and inclination have been found to play an important role on droplet impact dynamics [43,44].

The effect of surface curvature on droplet shape can be interpreted by examining the apparent contact angle of a droplet when impinging on a flat vs. a convex surface. A theoretical model developed by Wu et al. was applied to predict the apparent contact angle of the inkjet droplet on the curved cone surface [43]. For reasons of mathematical simplification, it is assumed that the droplet is deposited at the middle of the shaft length and the convex surface is part of a sphere of radius equal to the cone radius at that point (R = 250 μm). In addition, the surface is considered smooth. The intrinsic contact angle, which is a known constant for specific combinations of liquids and surface materials, was found in literature as θ_flat_ = 71° for a water droplet on an acrylic surface. Based on the Wu model, the apparent contact angle for water on the cone surface at the middle of its length is θ_convex_ = 77.8°. Figure 7a,b schematically illustrate the effect of surface curvature on contact angle and droplet shape.

It has been documented that by increasing the surface radius (reducing the curvature), the thickness of the film is decreased [45]. The aforementioned indicate that the film deposited on the curved cone surface is likely thicker than the respective one deposited on the pyramid. The variations may be minimal, however a distinct impact on the dissolution profiles is expected.

MN design introduces another significant parameter pertaining to the resulting coating morphology, which is surface inclination. Upon impact on a horizontal hydrophilic surface, the droplet stays put and oscillates and/or spreads into a film [46]. Oblique surfaces bring in the effect of gravity that, if the inclination is high enough, can activate non-uniform spreading, with the top contact angle to recede and the bottom to advance [47]. 

The inkjet coating methodology applied requires positioning of all MN arrays at 45° in respect to the horizontal plane, however the geometry of the individual designs alters the inclination of the droplet impingement surface. Figure 7c–d display the real inclination angle of the impingement surface for all MN designs. By default, cone and pyramid MNs create the same inclination angle, calculated at θ_cone_ = θ_pyra_ = 18.5°, whereas for the spear MNs the respective inclination is at θ_spear_ = 45°. Sahoo et al. conducted a study wherein visual evidence of the effect of the inclination angle on droplet spreading is provided [44]. For angles higher than 20° the droplet seems to slide and elongate downwards creating a bottom ridge. The phenomenon is intensified as the inclination angle increases. The conclusions of this study coincide with the SEM images of the coated MNs (Figure 7f–h). The film on the spear MN appears to be thinner with an intensified bottom ridge. In contradiction, the other MN design that features a flat surface but a significantly lower inclination angle (pyramid), bears a film of higher yet more uniform thickness.

Regarding the behaviour of the coating upon insertion, a respective study investigated whether the film adhesion to the MN surface was sufficient to prohibit shear-driven dislodgement during insertion. X-ray computer microtomography (μCT) was employed to visualise insertion of an inkjet-coated MN into eight plies of parafilm [22]. The experiments illustrated that the MN retains the coating upon insertion and there was no visual evidence that any material remained on the parafilm surface. The findings of this study provide a preliminary indication that the described coating regime (which was similar to the one applied here) results in robust films that withstand insertion without damage or dislodgement. Further evidence is expected to be drawn from the in vitro studies.

### 3.9. In Vitro Release Studies

**Figure 8:**
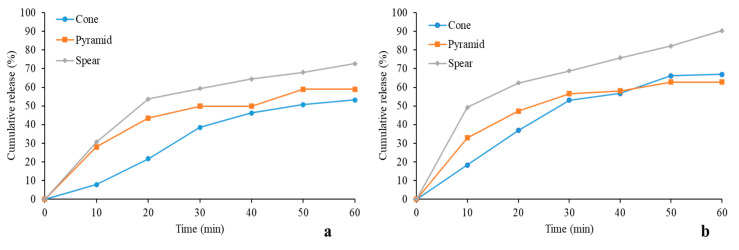
Cumulative release profiles for insulin delivered by 3D printed coated MNs with polymers as carriers; (**a**) poloxamer; (**b**) Polyvinylpyrrolidone (PVP).

The coated MNs were tested for their ability to deliver their insulin cargo in porcine skin. Figure 8 shows the cumulative release for all designs. PVP showed better performance overall in releasing the drug, owed to it being hydrophilic as opposed to poloxamer that features a middle hydrophobic block and two end hydrophilic blocks. Thus, a faster dissolution of the insulin-PVP coatings is justified. The sustained release of insulin due to the choice of the specific carriers served the purpose of this study, as rapid release generated by a different carrier is likely to have masked important effects, such as the dependence of coating dissolution on design. 

The highest drug revenue was delivered by the spear design for both polymer carriers, reaching up to 70% and 90% for poloxamer and PVP, respectively. Conversely, the cone design seems to have the least satisfactory performance for insulin-poloxamer, whereas its release for the PVP group improves. This is probably owed to the fact that the cone coating is likely bulkier and lags in releasing its cargo. This is further supported by the observation that for both pyramid and cone designs, the cumulative release was below 65% for 60 min. It is likely that the duration of the in vitro study was insufficient for complete coating dissolution for the two designs, in line with the above-described hypothesis of comparatively bulkier films, induced by geometry. It is anticipated that the entirety of the insulin cargo would be released by all designs for extended experimental duration, however additional data are required to verify this claim.

The results of this study support the hypothesis drawn earlier that MN geometry influences coating morphology in the described fashion, and thereby affects coating dissolution. In accordance with the geometrical explanation described earlier, drug release studies verified that the cone MN furnishes a surface prone to developing bulkier coatings, while the spear developed finer films. These findings are of high significance for applications in which gradual drug release is aimed at.

## 4. Conclusions

The application of 3D printing for MN manufacturing puts forward new challenges and calls upon the building of a new rationale behind MN design and development. The present study investigated the relationships between geometrical and manufacturing parameters with MN quality and performance, the knowledge of which is essential for tailoring and optimisation. 

The key conclusions of the study are:

1. Post-printing curing conditions influenced the mechanical properties of the material and MNs due to the degree of cross-linking. Optimisation with respect to the compressive properties is imperative to reassure the mechanical robustness of the MNs;
2. The printing angle affected MN quality and dimensional fidelity, with the tip to be the most prone to manufacturing defects;
3. MN geometry had an impact on piercing force, with cone-shaped MNs requiring the least force for skin insertion. Accordingly, geometry impacts fracture forces, with the spear-shaped MNs proving to be significantly weaker. All designs were found to be mechanically safe for application;
4. MN geometrical parameters such as surface curvature and inclination, influence coating morphology and dissolution, with spear exhibiting the best performance in releasing its insulin cargo in porcine skin.

Overall, we anticipate that the present study will contribute in elucidating critical problematic areas of MN 3D printing and advance knowledge in the field. 

## Figures and Tables

**Table 1:** Microneedle (MN) Computed Aided Design (CAD) dimensions.

	Pyramid	Cone	Spear
Length (μm)	1000	1000	1000
Base (μm)	1000 × 1000	Ø 1000	1000 × 80
Tip (μm)	100 × 100	Ø 100	100 × 80
Tip angle (^o^)	48.5	48.5	97
